# Querying and Extracting Timeline Information from Road Traffic Sensor Data

**DOI:** 10.3390/s16091340

**Published:** 2016-08-23

**Authors:** Ardi Imawan, Fitri Indra Indikawati, Joonho Kwon, Praveen Rao

**Affiliations:** 1 Department of Big Data, Pusan National University, Busan 46241, Korea; ardi@pusan.ac.kr (A.I.); fitri.indra@pusan.ac.kr (F.I.I.); 2 Department of Computer Science & Electrical Engineering, University of Missouri-Kansas City, Kansas City, MO 64110, USA; raopr@umkc.edu

**Keywords:** traffic sensor data, timeline model, historical traffic sensor data, TQ-index, traffic data query processing

## Abstract

The escalation of traffic congestion in urban cities has urged many countries to use intelligent transportation system (ITS) centers to collect historical traffic sensor data from multiple heterogeneous sources. By analyzing historical traffic data, we can obtain valuable insights into traffic behavior. Many existing applications have been proposed with limited analysis results because of the inability to cope with several types of analytical queries. In this paper, we propose the QET (querying and extracting timeline information) system—a novel analytical query processing method based on a timeline model for road traffic sensor data. To address query performance, we build a TQ-index (timeline query-index) that exploits spatio-temporal features of timeline modeling. We also propose an intuitive timeline visualization method to display congestion events obtained from specified query parameters. In addition, we demonstrate the benefit of our system through a performance evaluation using a Busan ITS dataset and a Seattle freeway dataset.

## 1. Introduction

Traffic congestion is one of the most prevalent transport problems in cities. Heavy traffic congestion can have many effects, such as delays, unpredictable travel times, increased fuel consumption, and road rage. For example, the total annual delay in travel time was around 13 billion people hours from the year 2010 to the year 2014, which consumed around six million tons of excess fuel in urban areas of the US [1].

Many countries use intelligent transportation systems (ITSs) to provide integrated road traffic-related services. ITS centers collect and store road traffic sensor data (occasionally, we use the terms “traffic sensor data” and “traffic data” interchangeably) from multiple heterogeneous sources, and mainly focus on providing real-time traffic information to alleviate the problems caused by traffic congestion. A Busan ITS center in South Korea captures traffic data every five minutes for each road link (road segment) and focuses on providing real-time traffic information. The traveler’s information website of Seattle provides real-time traffic conditions and travel times throughout the city.

Most traffic information services are based on data collected by ITS systems. PeMS [2], Highway England [3], Daum Map [4], Naver Map [5], and Here Map [6] offer more complete features based on ITS traffic data. In addition to ITS-generated data, several approaches also use crowd-sourced data to provide real-time traffic information [7,8,9].

Continuous historical traffic sensor data is generated once the real-time use of these data is complete. By utilizing archived historical traffic data, researchers find valuable insights, such as travel time predictions [10,11,12,13], traffic bottleneck analysis [14], and survival analysis [15].

While existing applications can efficiently provide limited analysis results, they are unable to cope with several types of analytical queries. In real life, there are many demands from citizens concerning traffic patterns on certain days or duration in traffic and changes in roads. For example, inhabitants of urban areas typically have analytical questions concerning road traffic behaviors, such as:

- $Q_{1}$: What types of congestion events occurred on Broadway Avenue (a road) last week?
- $Q_{2}$: Which road links are the most congested on a certain day?
- $Q_{3}$: Where is the heaviest congestion on a certain day?
- $Q_{4}$: If Broadway becomes congested, which other roads are affected?

This demand for specifically analyzed traffic information suggests the need for an interactive traffic query system which plays a key role in semantic applications for smart cities [16].

Motivated by these considerations, we investigate how to design an efficient road traffic analytical query system by exploiting historical traffic data. There are three critical challenges that arise in the design of such a system. They are: (1) how to model traffic information for interactive analytic queries; (2) how to facilitate the modeled traffic information for efficient query processing; and (3) how to provide intuitive visualization for the traffic query system.

**Figure 1:**
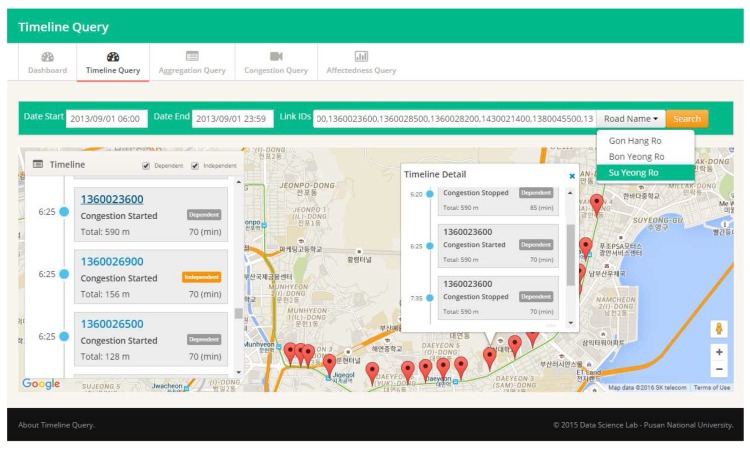
Traffic timeline features.

To address the above challenges, we propose a QET (querying and extracting timeline information) system, a novel system that facilitates effective analytical query processing based on a timeline model for road traffic sensor data. First, we devise an intuitive timeline model that can be used to capture the standard design as well as the spatio-temporal features of road traffic sensor data. In the QET construction phase, we extract timeline information from raw traffic sensor data by detecting the congestion events and obtaining some analytical information, such as an event’s dependency and the affectedness. Second, to enable efficient query processing over timeline information, we propose a TQ-index (timeline query index), which is an in-memory data structure that exploits spatio-temporal features of timeline modeling. We also develop several traffic analytical query processing algorithms that utilize the timeline modeling and TQ-index. Third, we suggest an intuitive timeline visualization method that displays congestion events of road traffic ordered by time along with the affected congestion lists and other useful analytical information, as depicted in Figure 1. Finally, we present the performance evaluation of the QET system using real traffic data such as a Busan ITS dataset [17] and a Seattle freeway traffic dataset [18]. Our QET system can handle different traffic data formats by converting raw traffic data and road network information into the timeline data model. The effectiveness of the TQ-index is measured by evaluating the query processing time and memory consumption.

This paper is an extended version of our previous publications [19,20,21]. The new contributions in this paper are as follows: First, we provide a complete formal timeline model for traffic congestion. Second, we present comprehensive algorithms for constructing the TQ-index and processing analytical traffic queries. In addition, we discuss the proofs of theorems to validate the correctness of the algorithms. Finally, we performed experiments to demonstrate the efficiency of the QET system. We conducted an extensive performance evaluation with real traffic data sets from Busan, Korea, and Seattle, WA, USA.

The remainder of this paper is organized as follows. Section 2 provides an overview of related work. We explain our background and motivation in Section 3. Then, we describe our proposed system and timeline modeling in Section 4. In Section 5, we present the detailed steps for constructing the TQ-index and explain how to utilize the TQ-index for processing analytical traffic queries. Section 6 discusses the demonstration and performance evaluation of our proposed system. We conclude our work in Section 7.

## 2. Related Work

Extensive research has been conducted in the area of traffic data analysis and management. The existing methods can be classified into the following categories: (1) traffic data analysis; (2) traffic data management and querying system; and (3) traffic data visualization.

### 2.1. Traffic Sensor Data Analysis

Because road traffic problems are prevalent in most countries, a variety of models exploiting historical traffic sensor data have been proposed for traffic sensor data analysis. Existing approaches can be broadly classified into three categories, namely (1) multi-dimensional spatio-temporal data approaches; (2) Markov model-based approaches; and (3) other approaches.

In the first category, several solutions have represented traffic data in time series as multi-dimensional spatio-temporal data modeling [10,22,23,24,25]. Lin et al. [22] proposed a novel floating car data analysis based on data cube modeling to explore traffic congestion patterns. Bajwa et al. [10] assumed that traffic patterns are recurrent within a tight time frame and proposed a travel time prediction model by considering matrices on a spatial and temporal scale in a pattern matching process. Lee et al. introduced a spatio-temporal congestion pattern to predict short-term traffic decongestion times [23]. A singular value decomposition (SVD) technique is used to reduce the traffic matrices to achieve an efficient query processing on road traffic data [24,25]. Most previous works focus on finding traffic patterns over different levels of spatial–temporal dimensions by aggregating congestion event data. We focus on providing a traffic query system that enables users to track the congestion events and the affected congestion events. In addition, we identify the starting point of congestion by measuring the dependency of the congestion.

In the second category, several traffic prediction systems are included [26,27,28]. Yang et al. [26] used spatio-temporal hidden Markov models (STHMM) to model correlations among different traffic time series. STHMMs focus on predicting near-future travel cost and cannot provide answers for the users’ queries. A 3D Markov random field was used in [27] to predict traffic flows, and uses both time series analyses and geometrical correlations. A HMM-based model [28] is proposed for urban-scale traffic estimation using floating car data. Traffic condition on a road segment is considered as a hidden state that can be estimated according to the conditions of road segments of similar traffic characteristics. Note that there is a big difference between the QET system and Markov-model based systems: QET aims to provide a novel analytical query processing method based on a timeline model for road; Markov model-based systems focus on providing the estimation of traffic conditions. Thus, Markov model-based approaches cannot provide answers for the users’ queries and cannot be directly applied to the QET system.

In the final category, there are also several approaches [11,12,13,29]. Chen et al. [11] developed a particle filter approach that used historical data for real-time short- to medium-term travel time prediction to select particles for partial resampling. A congestion-aware route planning system [29] is proposed to achieve a social optimum of all drivers’ travel time using historical taxi trip data and loop detector data. Li et al. [12] combined three data mining methods, such as K-means clustering, decision trees, and neural networks, to predict travel time with non-recurrent congestion. Elhenawy et al. [13] suggest a genetic program model that represents spatio-temporal interactions as algebraic expressions for the prediction of travel times. The result of these analytical processes provide a deep understanding of traffic behavior. However, these approaches do not support a query processing system for answering analytical questions, which is more useful to end users.

### 2.2. Traffic Management and Query System

As vehicular ad hoc networks (VANETs) have evolved, we can collect huge volumes of traffic information from On-Board Units (OBU) and various sensors of connected vehicles. A peer-to-peer (P2P)-based vehicular network technique [30], a mobile device-to-device video distribution scheme [31], and routing protocols in the Internet of Vehicles [32] help to collect multimedia traffic data such as images of license plates and videos of traffic congestions. In addition, the efficient distributed query processing platform [33] enables users to directly interact with the wireless network of traffic sensors. However, in this work, we focus on processing traffic sensor data which are collected and provided from current intelligent transportation systems.

Because of the accelerated growth of smart-phone technology, crowd-sourced data collection is now viable and can be implemented for traffic data processing domains. Google Traffic [7], Daum Map [4], Naver Map [5], and Here Map [6] use individual users’ locations to track real-time traffic congestion. Putu et al. [9] computed the degree of traffic congestion based on traffic information collected from SNS (social networking service) messages. Although there is an analysis process for obtaining real-time traffic information, these approaches do not provide deep insight into traffic behaviors. These systems also lack the capabilities to answer user queries (including timeline queries) about the analyzed information.

Most traffic data management systems use raw traffic data collected from intelligent transportation systems [34] which integrate information, sensor, control, and communication technologies [35]. According to the study of Kipileswar et al. [36], traffic management systems use ten types of sensing technologies. One of the technologies is an inductive loop sensing device, which has contributed to this work by providing freeway traffic data for Seattle [18]. DSRC (Dedicated Short Range Communications) has been used to obtain speed information from the Busan ITS center [17]. DSRC is a wireless communication technology developed for vehicular communication.

To effectively manage massive amounts of traffic data from sensors, one can use spatio-temporal query systems that have novel index structures developed by the database community [37,38,39,40]. Among these index structures, a timeline index is the most suitable index for managing temporal data [40]. This index structure is designed to support queries on temporal events. However, the structure should be extended to support spatial–temporal characteristics of road traffic information.

The most similar work to our QET system is a TransDec [41] system, which is a spatio-temporal query processing framework for transportation systems. TransDec offers a framework that enables real-time visualization, querying, and analysis of dynamic transportation. TransDec uses a Google Maps-based web application as a graphical user interface which allows users to formulate queries and provides query results. TransDec supports four types of queries: (1) monitoring queries on streaming data; (2) analysis and mining queries on historical data; (3) route planning queries; and (4) location-based queries. A monitoring query in TransDec is a continuous query which is issued once and is logically run continuously over the input streaming traffic sensor data. This query can report the speed information from highway sensor data in the map interface. To process analysis and mining queries on historical data, TransDec utilizes an online analytical query processing (OLAP) solution or wavelet-based techniques, depending on the size of datasets. The results are displayed as two-dimensional graphs on certain locations. A novel time-dependent route planning query is computed by using a time-dependent edge cost model for the networks. TransDec calculates different fastest paths from a source to a destination, depending on the time of the day. The result is displayed as a route in the Google Map-based interface. A location-based query of TransDec is a spatial query that looks for a desired point-of-interest to a referred object or location. A user might draw either a circle or a virtual monitoring boundary called GeoFence on the map interface, and TransDec provides results on the map.

There are several distinctions between TransDec and our QET system. First, TransDec uses a spatio-temporal database management system (DBMS) built on Oracle as a repository, whereas QET exploits a specialized data model and a novel index to maintain the timeline information of traffic behaviors. Second, QET can provide query functionalities on road traffic congestion events, along with the effects. Third, although TransDec supports various types of queries, it does not support timeline queries.

### 2.3. Traffic Data Visualization

Because of the complexity of traffic data and the number of features, several visualization methods have been proposed. An extensive survey of traffic data visualization methods can be found in [42].

One of the common road traffic information visualization methods is coloring the road network, depending on the situation, on a map [4,5,6,7]. Another method is to use a marker to denote an event that occurs at a point location [8]. Traffic origins [43] is another visualization method, which entails marking an area where the incident occurs. This approach allows us to observe the cascading effect of multiple incidents and the vehicle flow in the immediate vicinity. Unfortunately, all of these systems only bring limited traffic services because their crucial goals are to monitor current traffic conditions only. Thus, it is difficult to obtain chronological past information. Motivated by social network services (SNS) such as Facebook and Twitter [44,45], QET can show time-ordered traffic congestion events on a road network map.

## 3. Timeline Modeling

In this section, we first present the basic definition of road traffic data. We then describe two different traffic data sources that we use to verify our QET system. Finally, we explain the formal timeline modeling on which our QET system is based.

### 3.1. Traffic Sensor Data

Traffic sensor data refers to the datasets generated and collected by sensors in traffic vehicles or monitors installed along roads. These data typically include spatio-temporal properties and span substantial space and time. To reduce data size and for ease of subsequent analysis, the raw traffic data are aggregated for a particular time range. After providing the current status of existing roads, the real-time traffic data is archived as historical data, which can be analyzed in detail to explain the traffic behaviors. In this research, we used historical traffic data for Busan, South Korea and Seattle, WA, US provided by the ITS Centers of both cites.

#### 3.1.1. Definition of Traffic Sensor Data

To explain traffic sensor data in more detail, we provide the formal definition of crucial concepts used in our QET system. Typically, a city road network can be represented as a graph *G*, including a collection of nodes *V* and edges *E* (road segments) where each edge connects two nodes. A road segment is the smallest granularity in the road network (occasionally, we use the terms “road segment” and “road link” interchangeably), whereas a node can be located at an intersection or turning point where several road segments meet. In this work, every road segment is defined as a one-way road segment. Thus, each road segment has a source node and a destination node to illustrate the direction.

The formal definitions of a road and road segment are the following:

**Definition 1.**  (Road) Let $R_{i}=<r_{name},\left[n_{1},n_{2},n_{3},\cdots ,n_{k}\right]>$ denote a road in a city, where $r_{name}$ is its unique name in the city and $\left[n_{1},n_{2},n_{3},\cdots ,n_{k}\right]$ denotes a sequential list of nodes along the road.

**Definition 2.**  (Road network and road segment) Let a directed graph $G_{R_{1},R_{2},\cdots ,R_{i}}\;=\;\left(V,E\right)$ represent a road network of $R_{1},R_{2},\cdots ,R_{i}$, such that V is a set of nodes $n_{j}$ located in $R_{1},R_{2},\cdots ,R_{i}$ and E is a set of one-way road segments $L_{k}$ connecting two nodes. A road segment (in the following sections, we use the terms “road segments”and “road links” interchangeably) $L_{k}$ can be defined as $L_{k}=\left(lid,n_{from},n_{to}\right)$, such that lid is the unique identifier number for $L_{k}$ and $n_{from}$ and $n_{to}$ are the start node and the end node of $L_{k}$, respectively.

**Figure 2:**
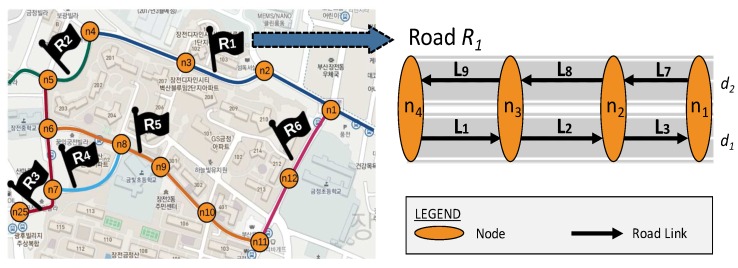
Road network model.

**Example 1.**  Figure 2 illustrates an example of a road network. This road network includes several roads, such as $R_{1},R_{2},R_{3},R_{4},R_{5}$, and $R_{6}$, which are printed in a different color. A circle denotes a node located in the road. For example, road $R_{1}$ contains nodes $n_{1},n_{2},n_{3},n_{4}$, and road $R_{6}$ contains nodes $n_{1},n_{12},n_{11}$. Consider a section of road network displaying the road segments including $L_{1}$, $L_{2}$, $L_{3}$, $L_{7}$, $L_{8}$, and $L_{9}$. Nodes $n_{1}$, $n_{2}$, $n_{3}$, and $n_{4}$ are connected with two road links in different directions $d_{1}$ and $d_{2}$.

We now formally define the notions of traffic sensor data. Although some devices create different types of information, such as speed, volume, occupancy, flow, or a combination of the preceding, we focus on the road network speed data.

**Definition 3.**  (*Traffic sensor data*) *Let $tr_{i,d,l_{id}}=v$ represent traffic sensor data, where lid is the identified road segment $L_{id}$, v is the speed value of the road segment $L_{id}$ at a certain time point on date d, and i is the $i_{th}$ sequence number of date d. A set of traffic sensor data $\left\{tr_{1,d,l_{id}},\;tr_{2,d,l_{id}},\;tr_{3,d,l_{id}},\;\cdots ,\;tr_{k,d,l_{id}}\right\}$ on the same date represents the daily traffic data of the road segment. If raw traffic data are collected and recorded every five minutes in a day, then the value of k becomes 288, which is computed by the equation* $\frac{1440}{5}$.

**Example 2.**  *Table 1 presents an example of traffic data from the same road link. From the second row, we find a road link ID (*1410046200*) and a speed value (*55*). After calculating the sequence of traffic data on 2016-02-14, we construct traffic data as $tr_{85,2016-02-14,1410046200}=57$. In the same way, we identify four traffic data as $tr_{86,2016-02-14,1410046200}=45$, $tr_{87,2016-02-14,1410046200}=48$, $tr_{88,2016-02-14,1410046200}=51$, and $tr_{89,2016-02-14,1410046200}=58$. Note that this table only displays a fragment of traffic data. The real traffic sensor data contain data from various road links.*

In the QET system, we use two different sources of historical traffic datasets to verify our methods. One is Busan ITS traffic sensor data, and the other is Seattle traffic sensor data.

#### 3.1.2. Busan ITS Traffic Sensor Data

The Intelligent Transportation System (ITS) Center of Busan provides historical traffic data. The data have been collected through DSRC sensors on Busan’s main arteries.

**Figure 3:**
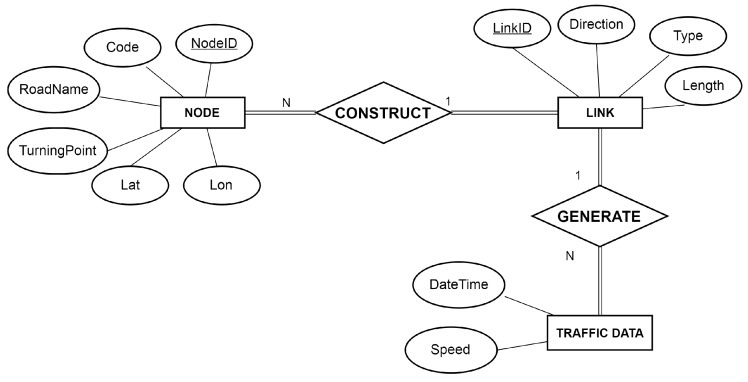
Entity-relationship (ER) diagram of Busan traffic data.

Figure 3 shows an entity-relationship diagram (ERD) of Busan traffic data. There are three entities: NODES, LINKS, and TRAFFICDATA. NODES and LINKS represent nodes and road segments in the road network defined in Definition 2, whereas TRAFFICDATA corresponds to traffic data defined in Definiton 3. The two relationships CONSTRUCT and GENERATE mean that each link consists of several nodes and can have several traffic data. Thus, we understand that the data format and the road network model of Busan ITS conforms to the formal definition of traffic data explained in Section 3.1.1.

#### 3.1.3. Seattle Traffic Sensor Data

**Figure 4:**
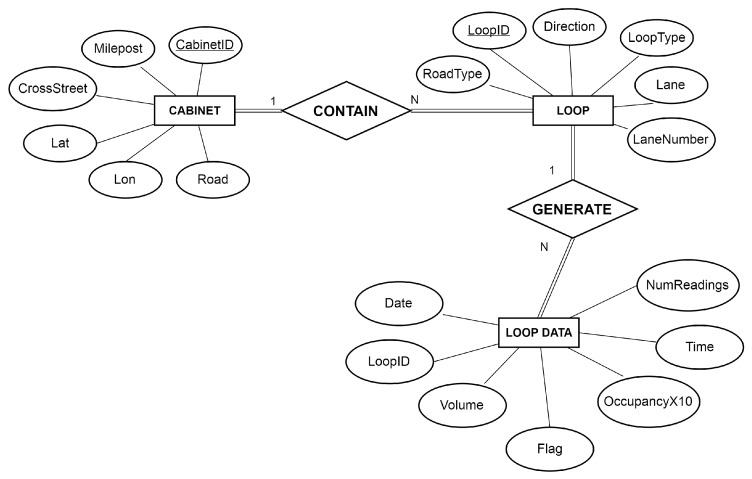
ER diagram of seattle traffic data.

Seattle traffic data were provided by Washington State Transportation Center (TRAC) [18]. In contrast with the Busan ITS data, the Seattle traffic data contain volumes and lane occupancies of road parts. Figure 4 illustrates an ERD of Seattle data with three entities and two relationships. Cabinet implies a single location point where several loop detectors are installed. Every loop detector records traffic conditions in each lane. Thus, a CONTAIN relationship is located between LOOP and CABINET entities, and a GENERATE relationship is located between LOOP and LOOP DATA entities.

**Figure 5:**
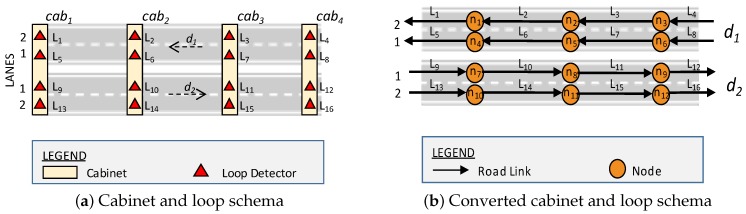
Road network of seattle traffic data.

To conform the Seattle dataset to the formal definition of road network 2, we convert the road network information. Figure 5a depicts an illustration of cabinets and loop positions. The cabinets are denoted by $cab_{1},cab_{2},cab_{3},cab_{4}$, where each cabinet contains several loop detectors. For example, $cab_{1}$ contains loop detectors $L_{1},L_{5},L_{9},L_{13}$ in different road lanes. The conversion was conducted by transforming a loop detector into a road link and setting LinkID to be the same as the LoopID. The last step is to create a virtual road segment that connects two road links. Figure 5b shows the converted road network of Seattle traffic data, which are consistent with road network Definition 2.

To conform LOOP DATA with the formal definition of Traffic Data 2, we use only Date, Time, LoopID, Volume, and OccupancyX10 attributes. Date and Time attributes represent a time point *t* on a certain day *d*. A LoopID denotes a road segment ID with the value of $L_{i}d$. Finally, we need to calculate speed value *v* from OccupancyX10 and Volume attributes by implementing the equation explained in [18].

### 3.2. Congestion

Traffic congestion is the most important information that we exploit in the QET system. Many different definitions of traffic congestion can be found in the literature. However, vehicle speed is most often used as a congestion indicator, since a large amount of vehicle speed data is easily detected by the loop detector, DSRC devices, and GPS from an urban road traffic system. In addition, vehicle speed reflects the travel behavior on an urban road network, and changes with the actual matching status of traffic demand and supply [46].

In QET, we define the traffic congestion as the road status based on the speed information. Associated with congestion, we introduce two concepts: (1) a congestion event that focuses on the change of status; and (2) the affectedness of the congestion in establishing the effects of the congestion. The formal definition of these concepts are the following:

**Definition 4.**  (*Congestion) A congestion $c_{k}$ is denoted as a four-tuple $c_{k}\;=\;<L_{id},t_{start},t_{end},type>$, where k is the unique identifier, and each $c_{k}$ means that a road segment $L_{id}$ has a traffic jam from time $t_{start}$ to time $t_{end}$ because its speed value is less than a given threshold value. The type of congestion will be determined after checking whether or not it is an affected congestion.*

**Definition 5.**  (Congestion event) A congestion event $e_{i,type}$ is denoted as a two-tuple $e_{i,type}\;=\;<L_{id},t>$, which describes that road segment $L_{id}$ has changed its $i_{th}$ status to either congested or decongested at time t. If the road segment starts to be congested or decongested, the event type of the congestion will be START or END.

**Example 3.**  *Consider again the raw traffic data in Table 1. We assume that a road link* 1410046200 *was not formerly congested, and the speed threshold for congestion is 55 km/h. The third row shows the congestion status for the first time because the speed is reduced to 45 km/h. We can consider this the START event of congestion. Thus, congestion $c_{1}=\left\{1410046200,2016/02/14\;06:05,-,-\right\}$ is created along with congestion event $e_{1,START}=\left\{1410046200,2016/02/14\;06:05\right\}$ if we assume this event is the first event of $c_{1}$. From the fourth and fifth rows, we find that the status of a congestion is maintained; thus, we do not create any events. The sixth row depicts a change in congestion because the speed value is higher than the threshold value. We generate a STOP event at that time point of the congestion. Thus, we can obtain a congestion event $e_{1,STOP}\;=\left\{1410046200,2016/02/14\;06:20\right\}$ and update $c_{1}=\left\{1410046200,2016/02/14\;06:05,2016/02/14\;06:20,-\right\}$. The fourth attribute of $c_{1}$ is explained in the next definition.*

Slow speed traffic data in a road segment may occur due to the same conditions of other adjacent road segments. Thus, we formally define an affectedness of a congestion.

**Definition 6.**  (Affected congestion) Given two congestions $c_{i}=<L_{i},t_{i1},t_{i2},type_{i}>$ and $c_{j}=<\;L_{j},t_{j1},t_{j2},type_{j}>$, congestion $c_{j}$ is an affected congestion of $c_{i}$ when the road link $L_{j}$ is the following (next) road link of $L_{j}$, and the start time $t_{j1}$ of $c_{j}$ is after (greater than) the start time $t_{i1}$ of $c_{i}$.

We classify a congestion as being one of two types, based on Definition 6.

- An independent congestion that occurs due to several factors, such as traffic lights, accidents, and infrastructure maintenance, and not because of other congestion. This congestion can be considered the head of congestion.
- A dependent congestion, which is an affected congestion.

If we consider a congestion path in which several road links are congested, we can identify the head (earliest) of the congestion that appears in the path. Because this congestion is not affected by the other congestion, it is independent. The remaining congestion in the path is affected congestion; thus, dependent.

**Figure 6:**
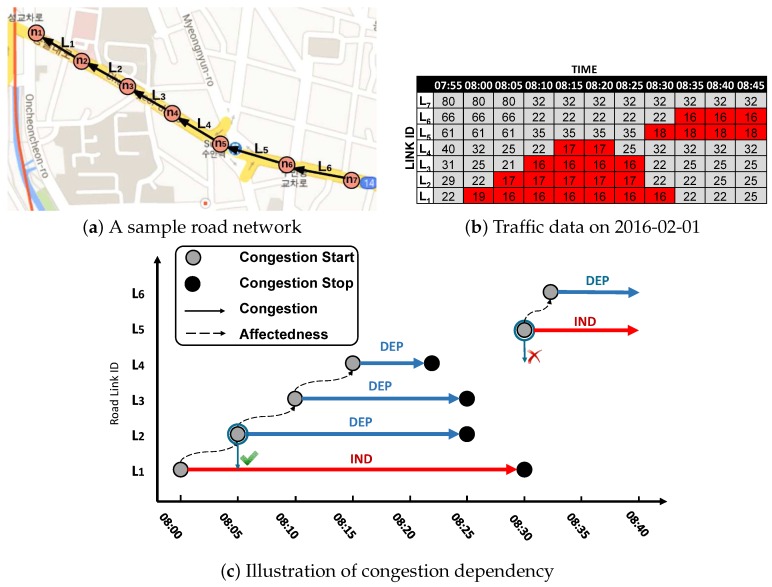
An example of congestion dependency.

**Example 4.**  Figure 6 illustrates the affectedness of congestion. Six road segments $L_{1},L_{2},L_{3},L_{4},L_{5}$, and $L_{6}$ are depicted in an example road network in Figure 6a. The table in Figure 6b contains speed information of road segments from $L_{1}$ to $L_{6}$ for every five minutes. The red cell in the table indicates congested road segments. The road networks in the top right show how these road segments are organized. Figure 6c explains how to understand the congestion dependency using a graphical view which is drawn by considering only values of red cells in Figure 6b. A gray circle and a black circle denote a START event and a STOP event of a congestion, respectively. Congestions in the path $L_{1},L_{2},L_{3},L_{4}$ and the path $L_{5},L_{6}$ are connected sequentially by a dotted line, representing the affectedness of a congestion. This happens because the next congestion occurs when the previous road segment is still congested. For example, a congestion of $L_{2}$ happens due to the previous congestion of $L_{1}$, illustrated by the green check mark. However, the congestion in $L_{4}$ and $L_{5}$ are not connected, because the START event of congestion $L_{5}$ occurs when congestion $L_{4}$ has stopped, illustrated by the red cross mark. The initial congestions in a congestion path can be considered independent congestions ($L_{1}$ and $L_{5}$), whereas the other congestions are considered dependent congestions ($L_{2},L_{3},L_{4}$, and $L_{6}$). As a result, the independent congestions and dependent congestions are depicted as a red solid arrow and a blue solid arrow, respectively, in Figure 6c.

### 3.3. Timeline Model

With previous definitions, we provide a formal definition of a timeline model, which is the main concept of the QET system in Definition 7. Intuitively, we try to connect a list of extracted congestions based on the start time and the affectedness. Thus, the timeline model enables us to describe the chronology and effects of congestion events.

**Definition 7.**  (Timeline model) A timeline model from raw traffic data is a set of $TM=\{tl_{i},tl_{2},\cdots ,tl_{n}\}$, where each $tl_{i}$ is represented by a pair of (congestionlist,eventlist) such that:

- a “congestionlist” is a sorted list of tuples ($c_{i}$, $p_{affectedby}$, $p_{affecting}$, dur, len) according to the start time of $c_{i}$ ($c_{i}.t_{start}$);
- an “eventlist” is a sorted list of congestion events $e_{i,type}$ according to the time $e_{i,type}.t$.

The congestion list is ordered according to the start time of $c_{i}$ (denoted by $c_{i}.t_{start}$), and its elements are tuples of ($c_{i}$, $p_{affectedby}$, $p_{affecting}$, *dur*, *len*). $c_{i}$ denotes congestion defined in Definition 4. $p_{affectedby}$ and $p_{affecting}$ are pointers representing the affected/affecting congestion in Definition 6. A $p_{affectedby}$ pointer links to the source of congestion, and a $p_{affecting}$ pointer refers to the next congestion that occurs due to this congestion. A value of dur is calculated by subtracting $c_{i}.t_{start}$ from $c_{i}.t_{end}$, and a value of len represents the total length of the congestion. An element of an event list is a congestion event $e_{i,type}$, as defined in Definition 5.

**Figure 7:**
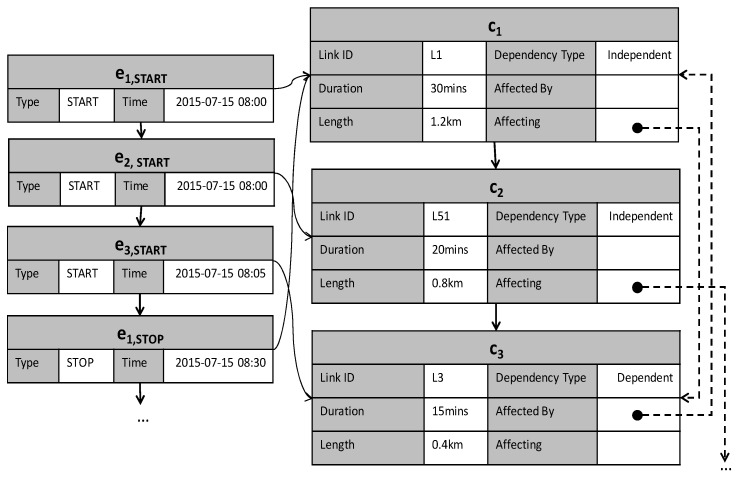
Timeline model.

**Example 5.**  Figure 7 depicts an example of the timeline model. A congestion list on the right is connected with an event list on the left. Three congestions $c_{1},c_{2},c_{3}$ are maintained in the congestion list. Because each congestion has a beginning event and an end event, it is connected to, at most, two elements of the event list. For example, congestion $c_{1}$ is connected to $e_{1,START}$ and $e_{1,STOP}$. Congestion $c_{2}$ is connected to only $e_{2,START}$, which means the congestion is still in progress. Congestion $c_{3}$ occurs due to congestion $c_{1}$. Thus, the $p_{affecting}$ pointer of $c_{1}$ is connected to $c_{3}$, and the $p_{affectedby}$ pointer of $c_{3}$ is connected to $c_{1}$.

## 4. Architecture of QET

In this section, we present an overall architecture of QET, which aims to achieve the following design goals:

- to exploit a timeline data model from different sources of traffic data;
- to leverage the power of the TQ-index to efficiently process “traffic analytical queries” using QET; and
- to provide high-level intuitive visualization to general users to aid understanding of traffic behaviors

**Figure 8:**
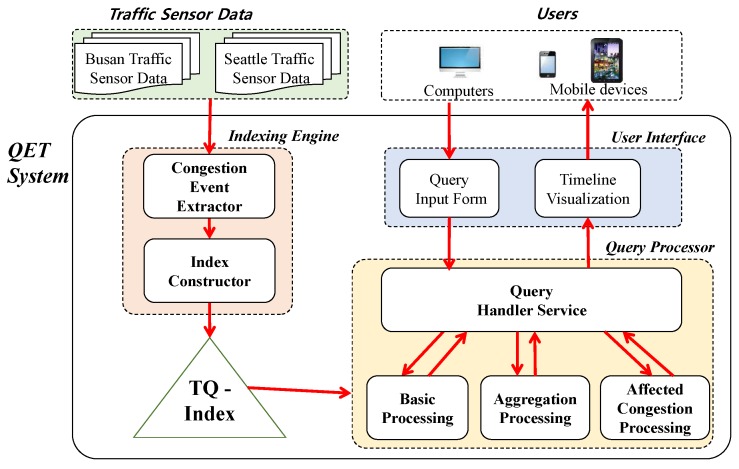
Key components of the QET system

We introduce the overall architecture of QET before delving into its details later in Section 5. The key components of QET are the Indexing Engine, the TQ-Index, the User Interface, and the Query Processor, as shown in Figure 8. The Indexing Engine extracts congestion events from the raw traffic data from Busan and Seattle, and stores them in the TQ-index. The TQ-Index is the heart of the QET system and serves as an effective spatial–temporal index for timeline information. Users of personal computers and/or mobile devices can identify their interests and obtain answers of timeline visualization through the web-based User Interface of QET. The User Interface consists of two sub-modules: (1) the Query Input Form; and (2) the Timeline Visualization module. The Query Input Form accepts a query from a user and passes it to the Query Processor of the QET system, and the Timeline Visualization module provides an efficient and intuitive visualization method for query responses. The Query Processor communicates with the User Interface through the Query Handler Service sub-module to obtain user queries or return query results. It also executes different types of analytical queries through the TQ-Index by invoking appropriate query processing sub-modules such as Basic Processing, Aggregation Processing, and Affected Congestion Processing.

The QET system allows any raw traffic data to detect congestion by considering slow-speed raw traffic data based on the timeline modeling. As explained in Section 3, the QET system extracts the congestion information that is represented as lists of START or STOP events of congestion. The system also analyzes the congestion more deeply to find hidden information, such as event dependency, affected congestion, duration, and the length of a congestion. All of this information will be elements of the timeline model for traffic congestion. The purpose of the TQ-index is to maintain the elements of the timeline model and exploit them for efficient analytical query processing.

QET supports different types of queries, which can be from specified users, and handles them in the Query Processor. We categorize an input query into three types: (1) a basic query; (2) an aggregation query; and (3) an affected congestion query. A basic query, such as $Q_{1}$, retrieves a set of congestion events according to the query parameters by utilizing the TQ-index. An aggregated query, such as $Q_{2}$ and $Q_{3}$, returns the aggregated information for further refinement steps. An affected congestion query ($Q_{4}$) aims to find the effects of a congestion that could be obtained by exploiting the TQ-index.

Because QET supports three different types of query, QET has several sub-modules for query processing. The Query Handler Service of Query Processor checks the validity of the input query, and is responsible for choosing appropriate query processing sub-modules according to the input query’s goal. The Basic Processing sub-module is a crucial part of query processing that exploits the TQ-index to quickly find all elements of the timeline model congestion for selected locations and time ranges. In addition, the result of this sub-module can be further refined to support other types of query. The Aggregation Processing sub-module includes the refinement steps after the Basic Processing sub-module. This sub-module consists of three steps: (1) grouping the basic query result based on a defined key; (2) calculating the aggregated value of grouped data; and (3) sorting aggregated data by the defined key. The Affected Congestion Processing sub-module tracks the affected congestions from the results of the basic sub-query processor. This sub-module traces all other congestions affected by any congestions found in the basic query. We shall provide the detailed procedures of these sub-modules in Section 5.3.

The User Interface of the QET system provides easier specification of an input query and employs timeline visualization for the results of the user queries. Figure 1 shows a screen shot of the User Interface of QET. The Query Inform Form enables a user to specify the parameters of an input query: road link(s), to determine a set of road links that should be investigated; and a start date and end date, to limit the time range of the input query. The user-specified queries are transferred to the Query Handler Service of QET via HTTP protocol. The Timeline Visualization module, which is implemented as a map-based web interface, provides the query results to give an intuitive user experience. Road link markers showing the results are displayed on the map. Thus, users can interactively access the timeline information by clicking the marker.

With this high-level overview of the system, we now explain the process of TQ-index construction and processing of the analytical traffic queries.

## 5. Timeline Query Index and Analytical Query Processing

In this section, we present our novel TQ-index scheme for maintaining timeline information converted from historical traffic sensor data. We first describe the TQ-index structure and the construction of algorithms from raw traffic data, and then explain how the TQ-index can be used to process traffic analytical queries.

### 5.1. The Index Structure

**Figure 9:**
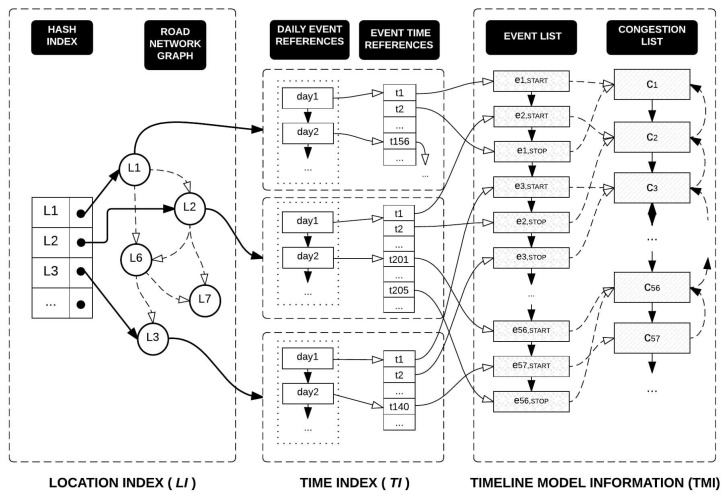
TQ-index.

Let $TM\;=\{tl_{1},tl_{2},\cdots ,tl_{n}\}$ denote the timeline model extracted from the historical traffic sensor data. As defined in Section 3.3, an element of the timeline model $tl_{i}$ mainly represents traffic congestion information with spatio-temporal characteristics. The TQ-index consists of three key components: (1) LocationIndex (denoted by LI) representing road networks; (2) TimeIndex (denoted by TI) for fast access to daily congestion events; and (3) TimelineModelInformation (denoted by TMI) to maintain the timeline model of the road network. Figure 9 depicts the overall design of the TQ-index.

**Figure 10:**
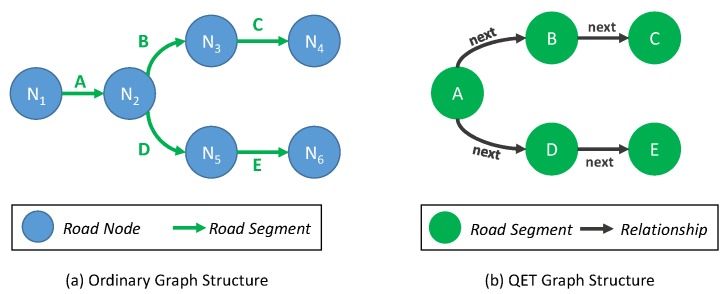
Road network.

LI contains a hash-based index and a graph representation of road networks, as illustrated in the left part of Figure 9. Because we focus on road segments as a spatial property of traffic information, we slightly modify the general concept of the graph representation as depicted in Figure 10. A node of a graph represents a road segment, whereas an edge of the graph illustrates a relationship between two road segments. The main reason for this modification is to optimize the tracing algorithm, because obtaining the next node is easier than obtaining the next edge in our approach.

To facilitate fast access to the specific road segments among abundant road links, a hash-based index is provided as a crucial component of location index LI. A hash key means an individual road segment and the value of the hash in a pointer to a node in the road networks. By following the next and previous pointers, we can identify the adjacent road links. In addition to the spatial information of the road network, a node on the graph also has a pointer to TI.

TI enables us to quickly locate time-specified congestion by checking the first event on that day and scanning the local events linearly. Thus, each element of TI is connected from each vertex of LI. TI mainly consists of two linked lists: (1) a daily event reference (DER) list and (2) an event time reference (ETR) list. The DER is a list of nodes, and each node of DER represents a day’s information, which is connected to the node of a local event reference (LER) list. An ETR list represents all event times for a road link. Each element of the ETR list is connected to a node of TMI. By combining DER and ETR, we can effectively identify the daily congestion events of a road segment.

TMI stores the timeline model information of the road network. As explained in Definition 7, the TMI consists of two lists : (1) a CongestionEventList to store a set of congestion events; and (2) a CongestionList to include a set of congestions. Because both CongestionEventList and CongestionList will contain a huge volume of timeline model information, the TQ-index has two indexes (such as LI and TI) to quickly identify a specified congestion or congestion event information. This relation is denoted as pointers in Figure 9.

### 5.2. TQ-index Construction

Given the formal definition in Section 3, we now explain the implementation methods of the QET system. In the QET system, a TQ-index is constructed by inserting elements from the timeline model, which are converted from historical traffic sensor data using four phases: (1) building LI; (2) extraction of elements from the timeline model; (3) inserting the elements into a TMI; and (4) inserting the elements into a TI. In the following subsections, we explain the detailed steps of creating the TQ-index.

#### 5.2.1. Location Index Construction

The first phase is to construct a graph structure in LocationIndexLI to support various types of road networks. The LocationIndexLI maintains a road network as a spatial part of the collected traffic sensor data. In our QET system, LI consists of a HASH INDEX and a ROAD NETWORK GRAPH. For each road link, we create a vertex into ROAD NETWORK GRAPH of LI. Then, we insert a new element to HASH INDEX with lid as the key including a pointer to the vertex in ROAD NETWORK GRAPH. Each vertex of ROAD NETWORK GRAPH has two pointers that connect to the next vertex and the previous vertex, which represent a link of a graph structure. By accessing each element in HASH INDEX and following the pointers of vertex in ROAD NETWORK GRAPH, we can quickly identify adjacent road segments. Algorithm 1 outlines the steps in constructing LocationIndexLI.

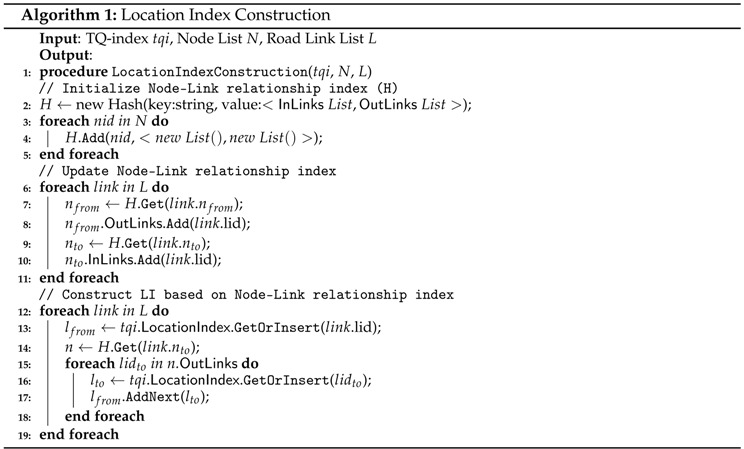

**Figure 11:**
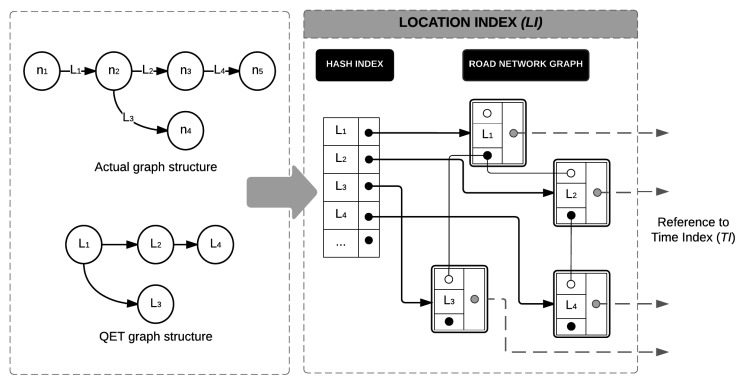
Location index.

**Example 6.**  Figure 11 illustrates how LI is constructed from a road network in the top-left part of the figure. The road network consists of five nodes $n_{1},n_{2},n_{3},n_{4}$, and $n_{5}$, and four links such as $L_{1},L_{2},L_{3}$, and $L_{4}$. As explained in the previous subsection, we used the modified road network as shown in the bottom-left part of the figure. The node of the QET graph structure represents four road segments $L_{1},L_{2},L_{3}$, and $L_{4}$, and there are four elements in HASH INDEX. Element $L_{1}$ points to the vertex $L_{1}$ in ROAD NETWORK GRAPH. The vertex $L_{1}$ has a next pointer that connects to $L_{2}$, and vertex $L_{2}$ has the previous pointer connected to vertex $L_{1}$. A next pointer is represented as a solid black circle, whereas a previous pointer is represented as an empty circle. Because a road link could be connected to more than one road link, it might have multiple points, such as $L_{1}$.

#### 5.2.2. Extracting Elements of the Timeline Model

This phase discovers elements of a timeline model that includes congestions and congestion events, along with the affectedness that is extracted from raw traffic data. This timeline model corresponds with Definition 7. This process is conducted incrementally, as we would read daily data. Raw traffic data is regarded as a set of three-tuple (linkID, datetime, speed) as explained in Definition 3. This information is written as a line of text in a real traffic log file. The daily data are stored in several log files. Thus, first, we read all daily log files and then sort them by time to obtain the effects of congestion over time. This extraction phase operates in two steps to obtain daily traffic congestion events. The subsequent subsections provide the detailed explanation.

#### Congestion Event Detection

For this step, we detect a START event (the beginning of congestion) or a STOP event (the end of congestion) of congestion explained in Definition 5

**Figure 12:**
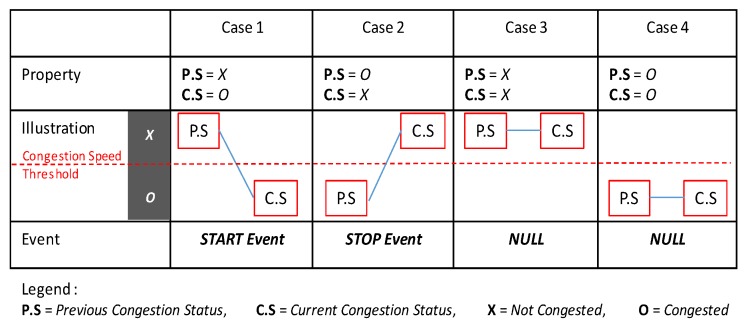
Case for congestion events detection.

The congestion events detection in Algorithm 2 begins by reading daily raw traffic log files *f* and initializing a set of daily events *E*. From line 4, we start to detect congestion events for each traffic data record. Lines 5 to 6 and 12 to 13 identify the change in congestion by comparing the current congestion status with the previous congestion status. There are only four cases of congestion status comparison among all of the raw traffic data, as illustrated in Figure 12. Case1 occurs when the congestion status moves from not congested to congested, and Case2 occurs when the congestion status moves from congested to not congested. However, Case3 (Case4) implies the maintenance of a congested (decongested) state. From Case1, a new START event would be produced as written in Lines 7 to 8, and the current status will change to congested in Line 10. In contrast, Case2 produces a STOP event as depicted in lines 14 to 15, and the current status will change to not congested. In both cases, a new congestion event will be added to *E* in lines 9 and 16. Nothing is done for Case3 and Case4, because they maintain the same state.

We now state the correctness of Algorithm 2. The following proposition is key to establishing the correctness of algorithm Congestion Events Detection.

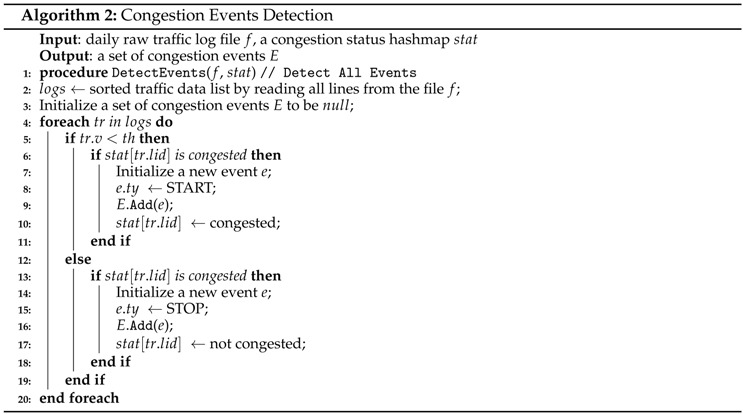

**Proposition 1.**  Given a road link l, the congestion event is one of the cases in Figure 12. If we compare the previous status and the current status of road link l, it is easy to find that the four cases are the possible combinations of the two statuses.

**Lemma 1.**  For road link l in the road networks, we can have either a START event or a STOP event if the status of l is changed.

**Proof.**  By Proposition 1, a road link *l* can have only one of the four cases. However, only Case1 and Case2 create a new START event or a new STOP event, because these cases imply a change in congestion status. Case3 and Case4 do not generate any events (NULL), because these cases maintain the same state as the possible state of road link *l*. ☐

**Theorem 2.**  [Correctness of congestion event detection] Congestion Events Detection algorithm is correct. In other words, for any input of raw traffic log f, the algorithm terminates after obtaining a set of congestion events E by considering only status changes of congested road segments.

**Proof.**  Before the loop in line 4 is executed, stat of all road links is notcongested, logs is sorted by time, and *E* is an empty list. stat can be considered a previous congestion state of all link ID. For every iteration, the current congestion state can be obtained by checking the log.speed value. If a value of log.speed is less than the threshold th, then the current state is congested; otherwise, it is not congested. By Lemma 1, it is true that the first detected event of all road links is a START event, because we set notcongested as the initial status, and a STOP event will be detected later. This proves the correctness of the Congestion Events Detection algorithm. ☐

#### Congestion Dependency Calculation

The congestion events extracted by Algorithm 2 are further examined for the property of congestion dependency. This is because road link congestion will affect subsequent congestion of the following road links, or it is an affected congestion caused by the previous congestion of other road links. The type of dependency can be utilized to indicate that specific congestion is the head of congestion in road links or the affected congestion. In addition, we also connect related congestion to reveal the effect of the congestion.

Algorithm 3 shows the process for revealing the affectedness of a congestion event. To identify affectedness, we check the congestion status of the adjacent road link for the given congestion event. Thus, an iteration of the checking will be done for a set of previous road links and a set of subsequent road links in lines 4 to 9 and lines 10 to 15. If prior congestion exists in the previous link at the same time in line 4, this implies that the congestion event has occurred due to congestion in the previous road link. Thus, the dependency type of the congestion is set to DEP (dependent). In other cases, the dependency type maintains the value of IND (independent), which is initialized in line 3. Whenever simultaneous congestion occurs in the subsequent road links, the type of these congestion events is automatically set to DEP in line 12. In lines 6 to 7 and 13 to 14, we connect related congestion events to construct a congested path (several road links) and see the effects of the congestion path.

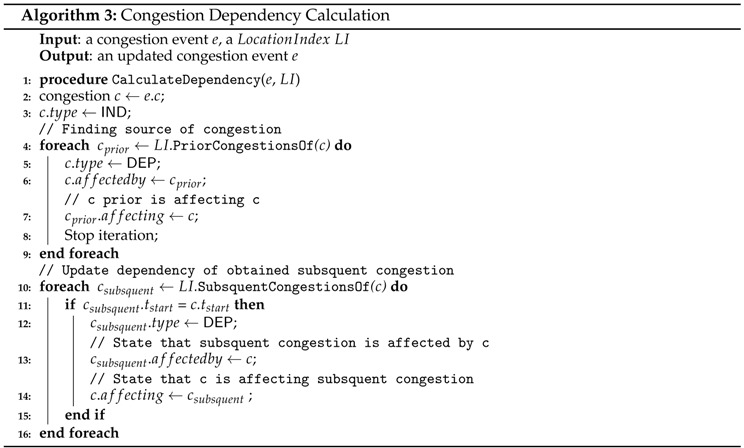


We discuss the correctness of Algorithm 3 using the following theorem.

**Theorem 3.**  [Correctness of congestion dependency calculation] The Congestion Dependency Calculation algorithm is correct. In other words, for a congestion path containing n road links, every road link in the path can have either DEP or IND as the type.

**Proof.**  As shown in Figure 6a, a congestion path can have several road links. We prove the lemma by induction on the number of road links *n*. Let P(k) denote the congestion path of k road links and L(k) denote the $k_{th}$ road link in P(K).

(1) Basis of induction: P(1) is true. Generally, L(1) means a single road link. It is typically the head of the congestion path, and the type is set to IND.
(2) Induction hypothesis: Assume that P(i) is true for 1≤i≤k. We show that P(k+1) is true. The road links in P(k) are connected because the congestion events L(2),L(3),⋯,L(k) are dependent.

Case 1: If the congestion in road link L(k+1) is an independent IND, then it could be a starting node of a new congestion path $P^{\prime }\left(1\right)$ and not a road link of P(k+1).

Case 2: If the congestion in road link L(k+1) is affected by the previous congestion in L(k), then it has dependent congestion (DEP) type, which means it can be connected to P(k). Thus, P(k+1) is true. ☐

#### 5.2.3. Insertion to Timeline Model Information (TMI)

At this phase, we construct the TimelineModelInformation TMI of a TQ-index from extracted congestion and congestion events. The extracted information obtained in the previous algorithms will be inserted into EventList (EL) and CongestionList (CL) of TMI. The elements of these lists are explained in Definition 7, and the structure of these lists is depicted in Figure 9. Algorithm 4 shows this insertion phase. We obtain the TimelineModelInformation TMI from the given TQ-index (line 2). Then, for each congestion event in *E*, we add this information to the EventList of TMI by invoking AddToEventList in line 4. Then, we add the congestion *c* of *e* to the CongestionList of TMI by calling AddToCongestionList in line 6. This addition is invoked only when the type of *e* is START. Otherwise, we do not add the congestion again into the CL. The last step is to connect the congestion event and the TimeIndexTI.

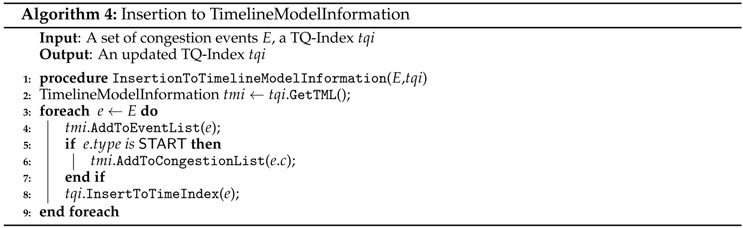


#### 5.2.4. Insertion to TimeIndex TI

For this phase, we build a TimeIndex (TI), which presents a temporal part of traffic data. The main components are DailyEventReferences (DER) and EventTimeReferences (ETR) explained in Section 5.1. TimeIndex (TI) enables us to efficiently access a set of congestion events maintained in the EventList of TimelineModelInformation (TMI). The complete process is explained in Algorithm 5. First, we find the related vertex node of LocationIndex (LI), because TI is located in each vertex of LI (line 2). Then, we create an event time reference element etr that keeps a pointer to a congestion event *e* and add it to ETR of TI. Next, we try to find a node in DER that contains a reference to the first event in ETR on that given day (line 6). If the node does not exist, then we create a new node and add it to DER with a given day as the key and then refer to etr (lines 7 to 9).

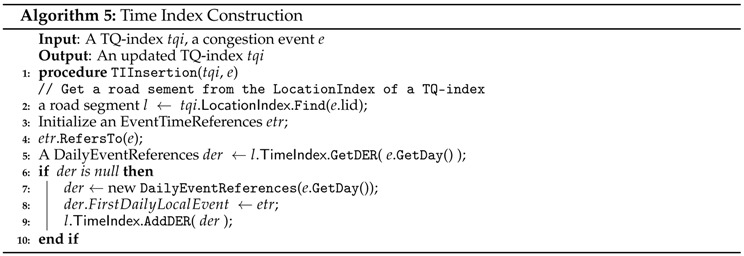


After the execution of this phase, we have completed the construction of all components of a TQ-index as depicted in Figure 9.

### 5.3. Analytical Query Processing

As explained in Section 4, QET supports three types of queries: (1) a basic query; (2) an aggregation query; and (3) an affected congestion query. In this subsection, we explain how to process queries by exploiting the structure of a TQ-index.

#### 5.3.1. Basic Query Processing

**Figure 13:**
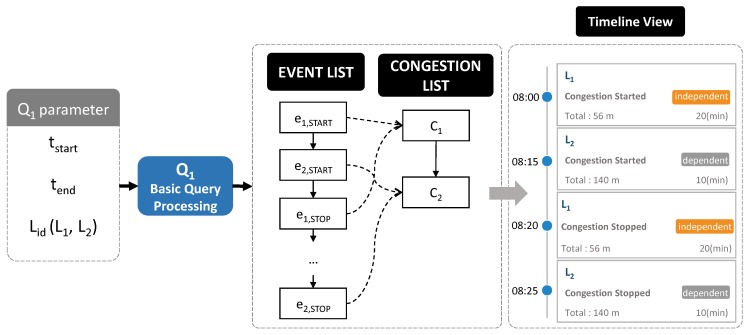
Basic query processing.

In this subsection, we present how QET exploits a TQ-index for the efficient processing of a basic query. Figure 13 depicts the overview of basic query processing. A user specifies parameters of a query $Q_{1}$ and returns the timeline model including information about traffic congestion in chronological order. This result is visualized as a timeline view in a web browser.

The basic query processing algorithm that identifies the congestion events in specified road links within the given time range is presented in Algorithm 6. A basic query utilizes the location index (LI) of a TQ-index to find specified road links from road networks (line 4). Then, the query obtains a time index (TI) of the location for locating the DailyEventReferences(DER) at the given start date (line 5). Once we obtain the starting event (line 6) , we can linearly scan the EventTimeReferences(ETR) through lines 7 to 11. While scanning, we collect a set of related traffic congestion and events that appear within the given start time and end time of the timeline model (line 9).

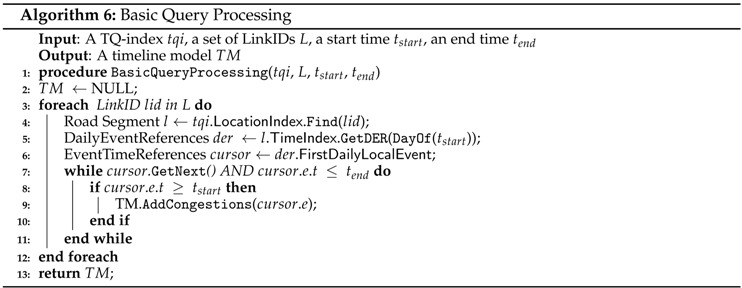

**Figure 14:**
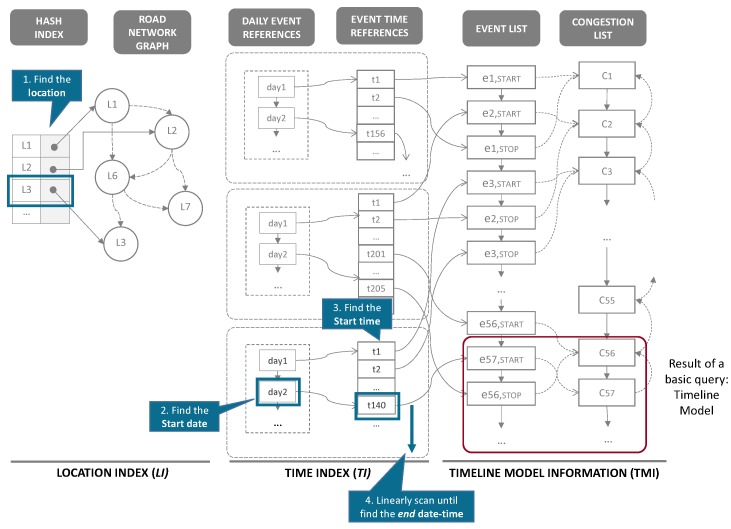
Basic query steps.

**Example 7.**  Assume that a user specifies identifying congestion events that occur in road link $L_{3}$ from day2 to day3. Figure 14 explains how a TQ-index is exploited during the basic query processing. First, we find the road link $L_{3}$ in LocationIndexLI (step 1). Then, following the pointer of $L_{3}$, we access the TimeIndexTI to locate the DailyEventReferences (step 2). In this case, we obtain the starting date point day2 from DailyEventReferences (step 3). From this list, we can start to scan linearly EventTimeReferences until we meet the time greater than day3. While scanning, we also collect a set of traffic congestion and events as a timeline model (step 4).

To prove the correctness of Algorithm 6, we use the loop invariant technique [47]. This approach examines the correctness of the algorithm in three loop stages: (1) initialization; (2) maintenance; and (3) termination.

**Theorem 4.**  With a given start time $t_{start}$ and an end time $t_{end}$ as input parameters of a query and a cursor as a pointer to trace EventTimeReferences, algorithm Basic Query Processing is correct with this loop invariant: for any step in the inner loop, this statement is applied: $t_{start}\;\leq \;$cursor.t $\;<\;t_{end}$, otherwise, the value of the cursor will be more than $t_{end}$ or it is NULL.

**Proof.**  **Inner Loop:** Before an iteration is started, a TQ-index tqi is already constructed, and LI is the LocationIndex of the TQ-index tqi. For each lid that is defined in the outer loop, the algorithm will find corresponding events that started between $t_{start}$ and $t_{end}$.

**Initialization:** When the iteration begins by exploiting the time index TI of tqi, cursor is supposed to be the first node whose time value (denoted as cursor.t) is between the given date range $t_{start}$ and $t_{end}$. Otherwise, cursor is supposed to be NULL.

**Maintenance:** Suppose that at the *i*th iteration, the cursor is at position *j* of LocalEventReferences and cursor.t of $cursor_{j}$ is still between $t_{start}$ and $t_{end}$. Then, because the long node at j+1 position exists and the cursor.t$<\;t_{end}$, we can update the cursor to position j+1.

**Termination:** Because cursor is a pointer that points the sorted elements of EventTimeReferences, there will always be the end of EventTimeReferences. At the end of the iteration, cursor could be NULL when it passes the end of EventTimeReferences, or the time of the cursor would satisfy the condition cursor.t$\;>\;t_{end}$ when it passed the matched elements of EventTimeReferences.

**Correctness:** At the end of the iteration (after termination), cursor would be NULL or the cursor.t, which is greater than $t_{end}$, trivially. ☐

#### 5.3.2. Aggregation Query Processing

An aggregate function is a function that needs a grouping of the values of multiple rows together to provide a single value. Our QET system supports the common aggregate functions, such as SUM, MIN, MAX, COUNT, and AVG for the traffic analytical queries. For example, query $Q_{2}$ in Section 1 needs to calculate COUNT values to find the amount of congestion on each road segment in a day and then apply MAX function to the count values. In this case, we use a road segment ID and date as the key for grouping. Another example is query $Q_{3}$ in Section 1, which needs to keep the longest duration of congestion in a day by applying the MAX function.

Algorithm 7 describes the detailed steps for aggregation query processing. This algorithm begins by obtaining a timeline model from basic query processing (line 2). Then, we retrieve a list of traffic congestion from the timeline model (line 3). There are two iterations in the next steps. The first iteration groups congestion list *C* based on the given key (lines 5 to 8). Then, the second iteration invokes AggregateFunction with a grouped set of congestion events as the parameter. The result of this function is aggregated values of the given set of congestion added into the aggregated value list (lines 10 to 13). Then, we sort the aggregated value list and return this list as the output of the algorithm.

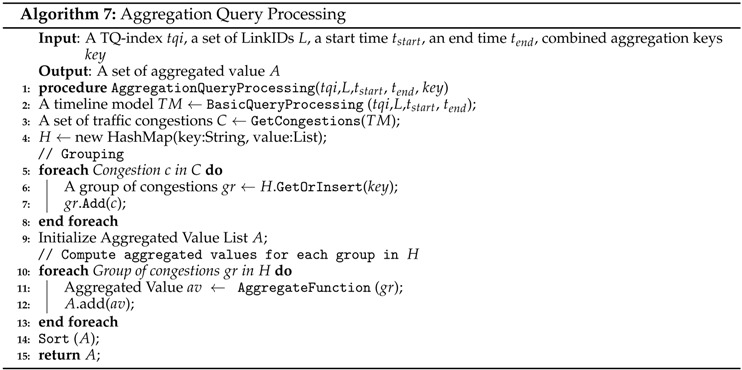

**Figure 15:**
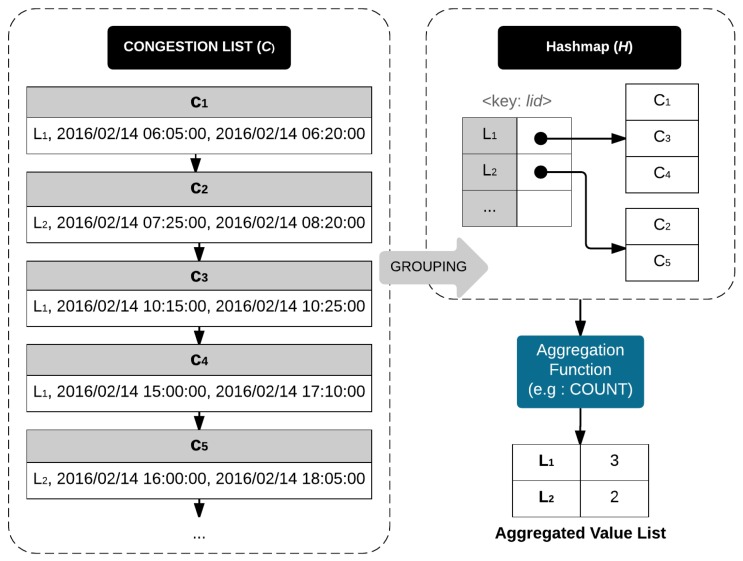
Aggregation query example.

**Example 8.**  Figure 15 describes aggregation query processing. We obtain a timeline model containing a list of traffic congestion events C by invoking BasicQueryProcessing. The elements of C are $c_{1}$, $c_{2}$, $c_{3}$, $c_{4}$, and $c_{5}$. Next, we apply the grouping to C to obtain hashmap H as a result. The Hashmap H contains only two elements: $L_{1}$$<c_{1},c_{3},c_{4}>$ and $L_{2}$$<c_{2},c_{5}>$. Then, we pass H into a COUNT aggregation function. The result shows the number of congestion events for each group in H. In this example, $L_{1}$ has three and $L_{2}$ has two congestion events.

The correctness of the aggregation query processing algorithm is proven by following a loop-invariant verification technique.

**Theorem 5.**  [Correctness of aggregation query processing] The aggregation query processing algorithm is correct with two given loop invariants: In the first loop, H is empty or contains a critical key; for the second loop invariant, the number of A is the same as the number of iterations.

**Proof.**  Before the iteration begins, assume that TM is the result of basic query processing, *C* is a finite set of traffic congestion from TM, and *H* is an empty hash map.

**Initialization:** (First Loop) At the beginning, *H* is empty, trivially.

**Maintenance:** (First Loop) For any steps in the iteration, if *H* does not contain a value of key as keys, then a new list will be inserted to *H* with the value of key as a key.

**Termination:** (First Loop) After the last iteration, we guarantee that *H* contains a value key as a key, which is explained in the maintenance part.

**Initialization:** (Second Loop) At the beginning of the loop, a step number *i* is initialized to 0, and *A* is still empty.

**Maintenance:** (Second Loop) For each iteration, the new value from AGGREGATION-FUNCTION will be included as an element of *A*. Thus, at the *i*th iteration step, when i>0, the number of *A* equals *i*. When we move to the next iteration step i+1th, a new value for the group gr will be added to *A* again, which leads to an increase in the number of elements *A* to i+1.

**Termination:** (Second Loop) At the end of the iteration, the value *i* is equal to the size of *H*, and the number of elements in *A* would be the same as *H*. Thus, the number of elements in *A* would be exactly the same as *i*.

**Correctness:** Both loop invariant methods are working with the finite sets. Thus, they are always terminated and produce the correct results. ☐

#### 5.3.3. Affected Congestion Query Processing

An affected congestion query $Q_{4}$ requires the exploitation of the affectedness list, which is built during the CalculateDependency algorithm. For the affected congestion, we proceed with the results of the timeline model, as shown in Figure 14.

Algorithm 8 explains the procedures. First, we invoke BasicQueryProcessing to obtain a timeline model. After obtaining a set of congestion events, we simply track the affecting pointers for each congestion’s node until we reach the last (lines 5 to 7).
**Algorithm 8:** Affected Congestions Query Processing **Input**: A TQ-index tqi, a set of LinkIDs *L*, a start Time $t_{start}$, an end time $t_{end}$ **Output**: A set of congestions ret1: **procedure**
AffectedCongestionsQueryProcessing(tqi, $t_{start}$, $t_{end}$, lid);2: A timeline model TM ← BasicQueryProcessing (tqi, *L*, $t_{start}$, $t_{end}$);3: C←
GetCongestions(TM);4: A set of affected congestions RET ← NULL;5: **foreach**
*congestion c in C*
**do**6: | **if**
*c.affecting is not NULL*
**then**
RET.Add( *c*.affecting);7: **end foreach**8: **return**
RET;

The correctness of the affected congestions query processing algorithm is proved by following a loop invariant verification technique.

**Theorem 6.**  [Correctness of affected congestion query processing] The affected congestion query processing algorithm is correct with a given loop invariant: At the start of the loop, the number of elements in RET is equal to or greater than the number of iteration steps.

**Proof.**  **Initialization:** (Second Loop) At the beginning of the loop, a step number *i* is initialized to 0 and RET is still empty.

**Maintenance:** For each iteration, the newly affected congestion from congestion *c* will be included as an element of RET. Thus, at the *i*th iteration step, when i>0, the number of RET equals *i*. When we move to the next iteration step i+1th, the new affected congestion will be added to RET again, which leads to an increase in the number of elements RET to i+1.

**Termination:** At the end of the iteration, the value *i* is equal to the size of *C*, and the number of elements in RET would be the same as *i*.

**Correctness:** This loop invariant method proves that the algorithm will be terminated and produce the correct results. ☐

## 6. Experimental Results

In this section, we present the performance evaluation of our QET system.

### 6.1. Environment

We implemented the QET system in JAVA language using JDK version 1.8 with the maximum JVM of 4096 MB. All experiments were conducted on a commodity machine equipped with an Intel Core i5-4460 3.2 GHz Quad-Core Processor and 12 GB of main memory. To obtain sound and reliable experimental results, we repeated every test 10 times and averaged all the reported experimental results over all of the repetitions.

### 6.2. Datasets

We used two real datasets in the evaluation of the QET system: (1) Busan ITS traffic sensor data; and (2) Seattle traffic sensor data. In subsequent discussions, these datasets will be referred to as “Busan” and “Seattle”. The “Busan” dataset was collected from September 2013 to September 2015. Although the collection period was 24 months, there are some missing data for October 2013, April 2015, and November 2014. We exclude those dates to perform valid experiments, because the missing data can affect the number of congestion events. The data were collected from more than 12,000 road links, where 288 records are collected from each link per day. The amount of daily raw traffic sensor data is slightly more than eight megabytes. The total size of Busan ITS traffic is 5.6 gigabytes.

The “Seattle” dataset is available from the Research Data Exchange [18] web site, which is a transportation data sharing system managed by the US Federal Highway Administration. The data were collected from the PeMS (Performance Measurement System), which utilized loop detectors to report volume and occupancy in fixed intervals. For our experiments, we used five-minute freeway data from Seattle which were aggregated from raw detector data. The total data size is approximately 5.2 gigabytes, ranging from May 2011 to November 2011.

### 6.3. Experimental Result

To show that our system is working well and feasible, we conducted some experiments including index construction and query performance. We also show a data comparison chart to show the efficiency of the TQ-index.

#### 6.3.1. Index Construction

In the first set of experiments, we evaluate the performance of the TQ-index construction. To evaluate the indexing performance, we measured (a) the total wall clock time to build the index, and (b) the index size for varying input data size. We varied the number of months from 8 to 24 in increments of four for the “Busan” dataset, and from two to six months in increments of one for the “Seattle” dataset.

As explained in Section 5, TQ-index construction includes four steps: (1) location index (LI) construction; (2) timeline model (TM) extraction; (3) insertion to timeline information list (Til); and (4) insertion to time index (TI). Table 2 and Table 3 summarize the performance results of these four steps.

First, as we expected, total construction time increases with increasing dataset size. This is mainly because the amount of traffic congestion also increases as we vary the dataset size. Another observation is that the construction of the “Busan” dataset takes much more time than the construction of the “Seattle” dataset. The size of the “Busan” data is slightly bigger than the size of the Seattle data. However, the “Busan” dataset contains many primary roads, which contain more traffic congestion, whereas the “Seattle” dataset only contains traffic information on freeways.

The costs for TM extraction and insertion into TI become dominant as the dataset size increases, while the cost for LI remains the same and the cost for insertion to TiL also increases. Note that the cost for insertion into TI for the “Busan” dataset increased dramatically, while the insertion cost for the “Seattle” dataset slightly increased. The types of roads included in the datasets could be one reason. The “Busan” dataset consists of primary roads and highways. In some cases, several roads can be connected by one node, which results in many neighboring road links. However, the “Seattle” dataset contains only freeways. It is sufficient to check the next and the previous road links of a particular road link. The other reason is the amount of traffic congestion. Typically, substantial traffic congestion occurs in primary roads compared to freeways. Thus, the cost of the “Busan” dataset is dramatically increased with the dataset size. Because we used the same road network, the construction time for LI does not change significantly and remains almost the same. The cost for insertion to Til is slightly increased, because we insert a set of events into the list after checking the START status of each traffic event. In this case, the number of events is slightly increased with the size of the dataset.

**Figure 16:**
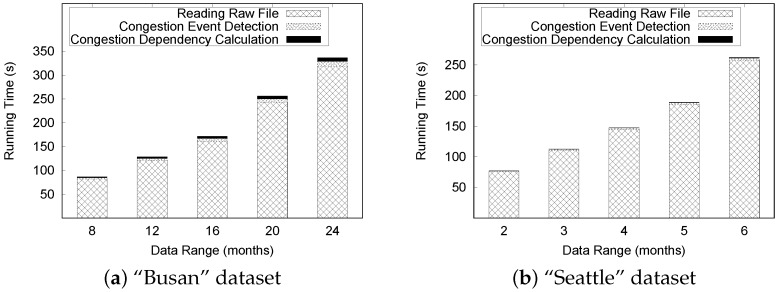
Construction time.

As we explained in Section 5.2.2, extracting the timeline model from raw traffic data is more expensive because of the disk I/O operations. Figure 16a,b show the detailed cost breakdown of TM extraction. The cost for calculating the congestion dependency becomes the dominant cost for both the “Busan” and “Seattle” datasets as we increase the data size, whereas the cost of reading raw traffic data is always the dominant cost. Detecting a congestion event occurs within a short time. As we expected, it takes a significant amount of time to read raw traffic data because this process requires disk I/O operations while the other steps are run in the main memory.

Calculating the congestion dependency status checking of all neighboring congestion to decide whether congestion is dependent. The cost for the “Busan” dataset increased dramatically, and the cost for the “Seattle” dataset slightly increased. The costs for this step could also be explained by the characteristics of the two datasets.

**Figure 17:**
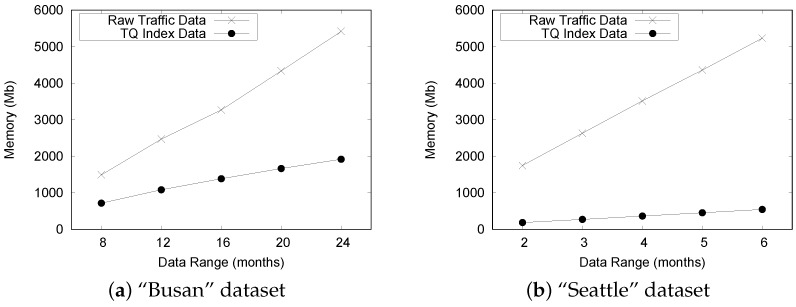
Size comparison between a TQ-index and raw traffic data.

Figure 17a,b show the effective reduction of memory consumption by comparing the size of the TQ-index with that of the raw traffic sensor data. The gaps in size widen as we increase the date range from 8 months to 24 months. We observe that the size of the TQ-index is much smaller than that of the raw traffic files in both datasets. This is because the QET system can effectively convert historical raw traffic sensor data into a smaller size TQ-index based on the timeline modeling. Another observation is that the TQ-index in Figure 17b reduces the raw traffic sensor data more effectively. The memory consumption of TQ-index for the "Seattle" dataset is small because it is collected from highways, which typically have less congestion than urban streets.

#### 6.3.2. Query Processing Performance Results

In this subsection, we analyze the query performance of the QET system that utilizes a TQ-index to maintain the timeline modeling.

We report experimental results for processing three different types of queries, as explained in Section 5.3. A user can specify three query parameters: (1) a set of road segments; (2) a start date; and (3) an end date. Thus, we conduct experiments with two settings: (1) a fixed date range setting; and (2) a varied date range setting. We randomly chose a contiguous five days between December 2013 and April 2014 (between May and August) for the fixed date range setting of Busan (Seattle), whereas we varied the length of the date range from 10 to 50 days for the varied date range setting. We randomly chose road links from three primary roads (SuYeong, Mandeok, GongHang) and two city highways (BeonYeong, Dongseo) for the “Busan” dataset. Similarly we randomly selected road links of the southbound and northbound freeway lanes for the “Seattle” dataset.

#### Basic Query Processing

**Figure 18:**
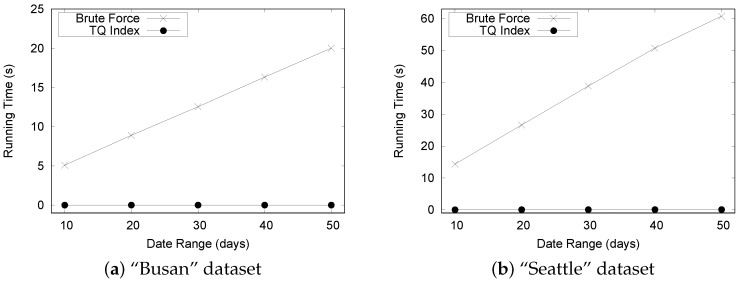
Brute force comparison.

In the first set of experiments, we evaluate the effectiveness of basic query processing by comparing our TQ-index method with a baseline approach. The baseline approach scans all raw files to obtain the results. Figure 18 depicts the experimental results by varying the date range. Clearly, it outperformed the baseline approach by utilizing the TQ-index. The execution time of the baseline approach increased with the date range, whereas the execution time of the TQ-index approach remained almost unchanged. The TQ-index enables us to efficiently locate the necessary congestion events due to the benefits of the timeline modeling.

Note that because the performance of the TQ-index is always superior to the baseline approach, we only report the performance of the TQ-index in the subsequent experimental results.

**Figure 19:**
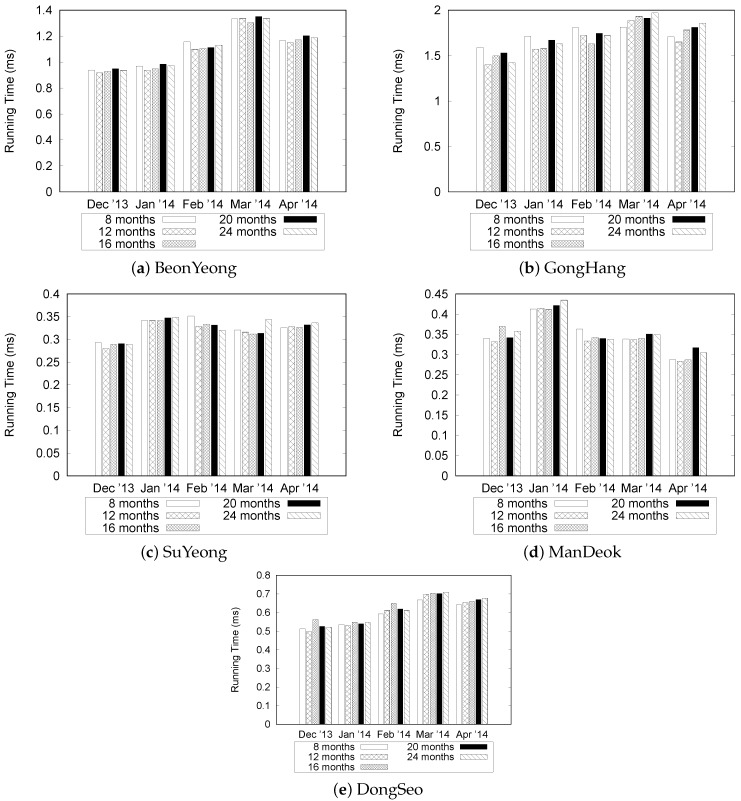
Fixed date range for the “Busan” dataset.

In the next set of experiments, we investigate the performance of the basic query processing under the various situations. First, we chose five different road links and randomly selected five consecutive days. Then, we varied the size of the “Busan” dataset from 8 months to 24 months. Figure 19 shows the results. We observed that the difference in execution time is less than 0.2 ms although we increased the size of data for BeonYeong in December 2013 from Figure 19a. For different months, the running time is slightly changed because we increased the data size, which demonstrates the efficiency of our QET system. From the graphs shown in Figure 19b through Figure 19e, similar trends can be observed from December 2013 to April 2014 at other road links. This is mainly because there are no significant differences in the number of events for the fixed date range. The results indicate that the TQ-index effectively processes the query by checking only relevant congestion events, regardless of the dataset size.

**Figure 20:**
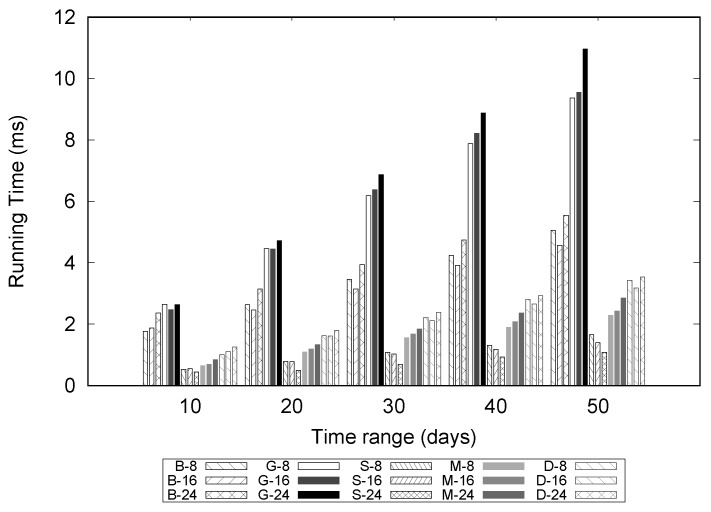
Varied date range for the “Busan” dataset.

Figure 20 shows the results for varying date ranges from 10 to 50 in increments of 10 days. The results for datasets with 12 and 20 months were omitted because they showed a similar trend to the datasets with 8, 16, and 24 months. In the graph, the notation “B-8” means that we use the eight-month data of BeonYeong road to construct a TQ-index. G, S, M, and D represent GongHang, SuYeong, ManDeok, and Dongseo, respectively. As expected, the execution time for GongHang (denoted by "G") increases linearly as we vary the date range. However, the execution times for BeonYong, Suyeong, Mandeok, and Dongseo do not increase considerably compared to the increases in the date ranges. Because the number of congestion events are increased with the date range, it takes more time to follow the CongestionEvnetList stored in the TQ-index. However, we can achieve effective query processing with the combination of LocationIndex and TimeIndex of the TQ-index. Another observation is that execution times for five roads in the “Busan” dataset are different. However, if we consider only the results of one road (such as G-8, G-16, and G-24), we see that the differences in execution times are small. This is consistent with the results in Figure 19.

**Figure 21:**
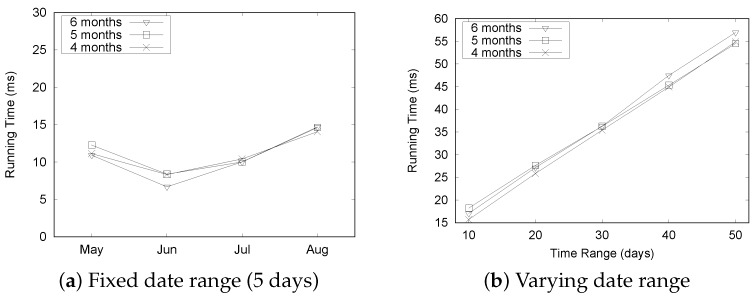
Basic query processing for the “Seattle” dataset.

Figure 21 summarizes the experimental results for the “Seattle” dataset. Although there is a slight fluctuation in execution times as depicted in Figure 21a, the query processing time remained quite stable as we increased the size of the dataset. The execution time increased linearly with the varying date range, as shown in Figure 21b. These results shows a similar trend to the “Busan” dataset and demonstrate the efficiency of our QET system.

#### Aggregation Query Processing

In this set of experiments, we present the performance evaluation results for processing the aggregation queries for the “Busan” and “Seattle” datasets.

**Figure 22:**
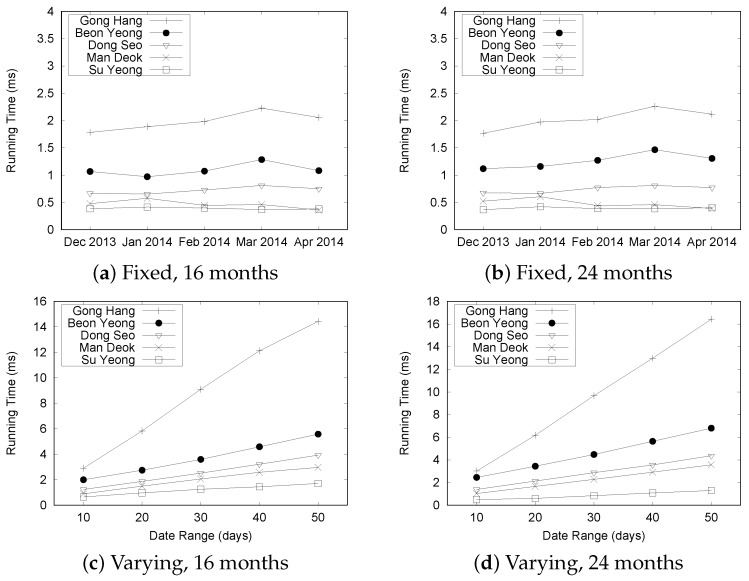
Query performance $Q_{2}$ on “Busan” dataset.

Figure 22 summarizes the results for processing $Q_{2}$ queries for the “Busan” dataset. The results from the data sizes of 8, 12, and 20 months were omitted because they showed trends similar to those from the data sizes of 16 and 24 months. For the $Q_{2}$ query type, a user specifies a start time, an end time, and road links as the query’s parameters to find the most congested road in a given time range.

Let us analyze the results shown in Figure 22a,b. In these experiments, we fixed the date range to five days. Because of the effectiveness of the TQ-index, the overall execution time is less than 2.5 ms in the fixed date range. This is mainly because we need to check only the pre-computed values of a TQ-index to process $Q_{2}$.

Figure 22c,d present the results under the varied date range setting. The query processing time for GongHang increases linearly as the number of dates increase, while the execution times for other roads increase slightly with the number of dates.

**Figure 23:**
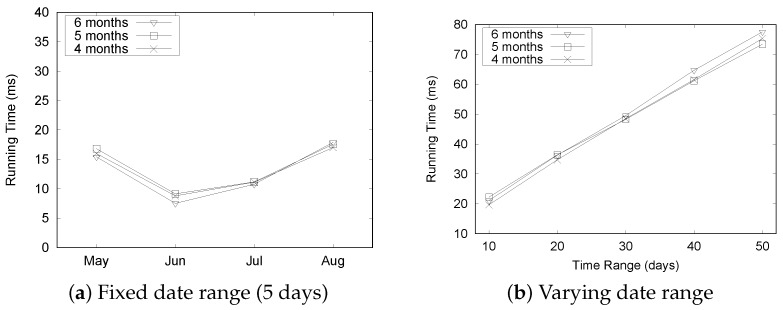
Query performance $Q_{2}$ on the “Seattle” dataset.

Figure 23 shows the execution time of the aggregation query $Q_{2}$ for the “Seattle” dataset. Figure 23a shows that there is little variation in execution times, but the overall time is less than 20 ms under the fixed date range setting. More time is required to process the “Seattle” dataset. The execution time for the fixed date range setting does not show significant change, even though we increase the size of the dataset. However, the execution time increases significantly with the number of dates, as depicted in Figure 23b. These results show the same trend observed in the case of the “Busan” dataset.

Next, we measure the performance of processing aggregate query $Q_{3}$. $Q_{2}$ computes the number of traffic congestion events, whereas $Q_{3}$ tries to find the longest congestion in a specified road link within a given time range. Thus, $Q_{2}$ requires a count function, and $Q_{3}$ represents a maximum function.

**Figure 24:**
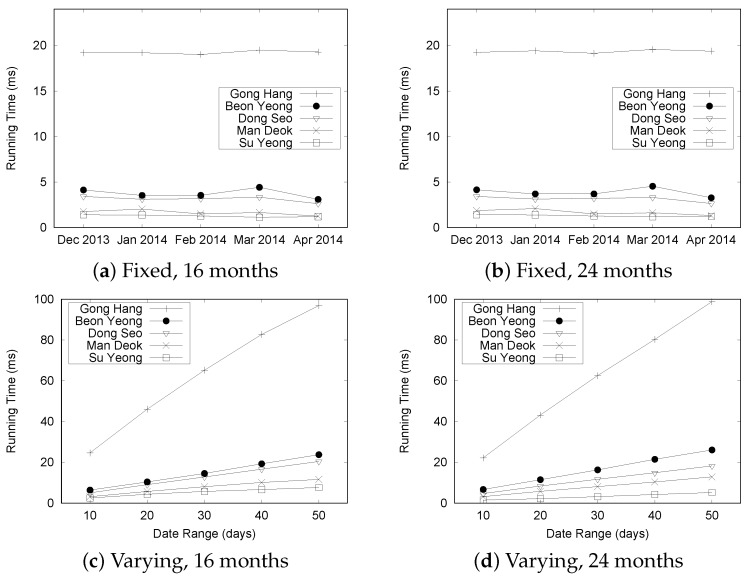
Query performance $Q_{3}$ on the “Busan” dataset.

Figure 24 depicts the results for the “Busan” dataset. As expected, the execution times of $Q_{3}$ are longer than those of $Q_{2}$, because we need to check adjacent road links to calculate the longest length of the congestion. Another observation is that the query processing time remains almost the same for the fixed date setting (Figure 24a,b), whereas the execution time is increased with the number of dates (Figure 24c,d). We explain the correlation between the number of traffic congestion events and the query processing time in another experiment.

**Figure 25:**
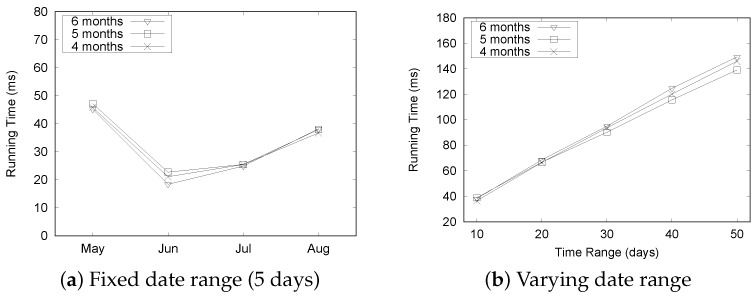
Query performance $Q_{3}$ on “Seattle” dataset.

Figure 25 shows the execution time of aggregation query $Q_{3}$ for the “Seattle” dataset. At the fixed date setting, the execution time is fluctuating as shown in Figure 25a. The longest execution time occurs in May, then suddenly drops in June and gradually increases again. This is explained by the fact that the different amounts of congestion events are substantial for each month. At the varied date range setting, the execution time increases linearly as we increase the number of dates. Again, these trends are consistent with the results of $Q_{1}$ and $Q_{2}$ for the varied date setting.

#### Affected Congestion Query Processing

**Figure 26:**
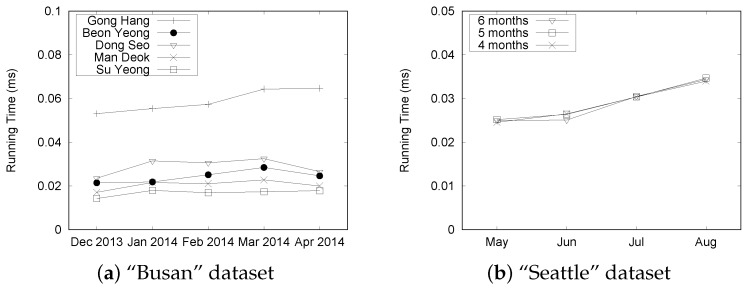
Query performance $Q_{4}$.

We investigate the performance of the affected congestion query processing. A user specifies a road linkID and a time of traffic congestion event as the parameters of $Q_{4}$. Figure 26 shows the results. Figure 26a,b show that only a small amount of execution time is required to process the affected congestion query. For the “Busan” dataset, the execution times for five different roads remain almost the same. The execution times for the “Seattle” dataset are also quite stable, without a significant increase, even though we increase the size of the dataset.

#### Varying Dataset Size

In this experiment, we analyze the performance of our QET system when we increase the size of the raw traffic sensor data. We set a date range of five days and select a date period from the 23rd day to the 27th day for each month. All road links for five roads of the “Busan” dataset and all road links of highways for the “Seattle” dataset are used in this experiment.

**Figure 27:**
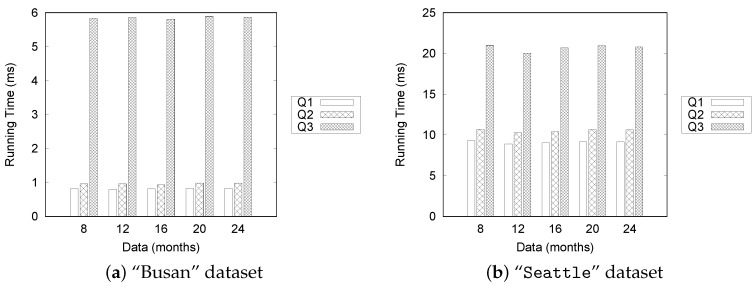
Varying dataset size.

The evaluation results for query types $Q_{1}$, $Q_{2}$, and $Q_{3}$ are summarized in Figure 27, and the results for $Q_{4}$ are described in Table 4. We find that it takes much less time (less than 0.1 ms) to process type $Q_{4}$ queries. Thus, we separate the results in the graph and the table.

In Figure 27, we observe that execution times for queries in the three types ($Q_{1}$, $Q_{2}$, and $Q_{3}$) remained almost the same as the dataset size increased. This can be explained by the efficiency of a TQ-index. We can effectively search LocationIndex and TimeIndex by taking user specified parameters, such as dates and road links. Another observation is that $Q_{3}$ always shows inferior performance for both the “Busan” and “Seattle” datasets. We need to calculate the length of each congestion event by checking all adjacent road links to identify the longest congestion specified by the query type $Q_{3}$. This additional computation causes significant performance degradation in the execution of query.

#### Effects of the Number of Traffic Congestion Events

**Figure 28:**
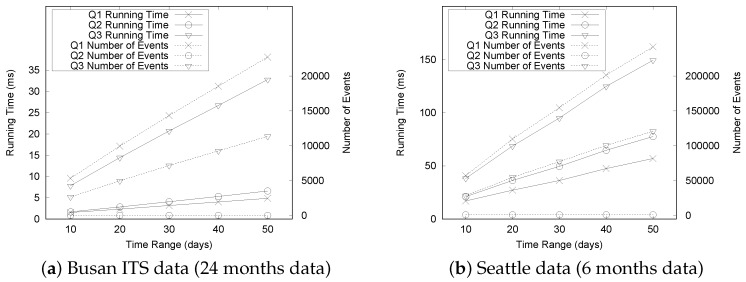
Event number and execution time comparison.

In the previous experiments, the query processing time increased with the date range. Figure 28 shows the correlation between the number of traffic congestion events and the execution time for $Q_{1}$, $Q_{2}$, and $Q_{3}$. We used all 24 months of data for the “Busan” dataset and all six months of data for the “Seattle” dataset. The trends in execution time follows the pattern of the number of traffic events. We think that the number of traffic congestion events can affect the query processing time.

## 7. Conclusions

In this paper, we presented a road traffic analytical query processing system called QET to query and analyze road traffic sensor data. For effective analytical query processing, the QET system employs: (1) a timeline model to extract traffic congestion events from raw traffic sensor data; (2) TQ-index to maintain the timeline model; and (3) efficient query processing algorithms to support the analytical queries. We conducted a comprehensive performance evaluation of the QET system using real datasets, Busan ITS sensor data, and Seattle freeway data. Our experimental results showed that the timeline modeling and the TQ-index of the QET system were effective in maintaining traffic congestion events and processing analytical queries by searching only relevant information.

## Figures and Tables

**Table 1:** Traffic log data.

LinkId	Date, Time	Speed (km/h)
⋯	⋯	⋯
1410046200	2016-02-14 06:00	57
1410046200	2016-02-14 06:10	45
1410046200	2016-02-14 06:15	48
1410046200	2016-02-14 06:20	51
1410046200	2016-02-14 00:00	58
⋯	⋯	⋯

**Table 2:** Building time for a TQ-index using Busan ITS data (s).

Month	LI Construction	TM Extraction	Insertion to TiL	Insertion to TI	Total Time
8	0.17	95.23	0.09	37.47	132.97
12	0.19	138.64	0.13	89.88	228.84
16	0.19	211.20	0.16	154.85	366.40
20	0.21	262.49	0.33	232.20	495.23
24	0.25	304.77	0.24	327.70	632.96

**Table 3:** Building time for a TQ-index using Seattle freeway data (s).

Month	TI Construction	TM Extraction	Insertion to TiL	Insertion to TI	Total Time
2	0.01	77.16	0.03	4.70	81.90
3	0.01	116.78	0.04	9.15	125.98
4	0.01	155.23	0.05	15.86	171.15
5	0.01	197.28	0.06	26.27	223.62
6	0.01	245.55	0.07	39.72	285.35

**Table:** (**a**) “Busan” dataset.

Month	Running Time (ms)
8	0.051
12	0.052
16	0.053
20	0.058
24	0.063

**Table:** (**b**) “Seattle” dataset.

Month	Running Time (ms)
2	0.028
3	0.026
4	0.029
5	0.031
6	0.033
